# For, against, and beyond: healthcare professionals’ positions on Medical Assistance in Dying in Spain

**DOI:** 10.1186/s12910-024-01069-1

**Published:** 2024-06-14

**Authors:** Iris Parra Jounou, Rosana Triviño-Caballero, Maite Cruz-Piqueras

**Affiliations:** 1 Department of Philosophy, Universitat Autònoma de Barcelona, Bellaterra, Spain; 2 Department of Public Health and Maternal-Child Health-Faculty of Medicine, Universidad Complutense de Madrid, Madrid, Spain; 3 Escuela Andaluza de Salud Pública, Granada, Spain

**Keywords:** Euthanasia, Assisted suicide, Medical Assistance in Dying, Bioethics, Relational autonomy, Qualitative study

## Abstract

**Background:**

In 2021, Spain became the first Southern European country to grant and provide the right to euthanasia and medically assisted suicide. According to the law, the State has the obligation to ensure its access through the health services, which means that healthcare professionals’ participation is crucial. Nevertheless, its implementation has been uneven. Our research focuses on understanding possible ethical conflicts that shape different positions towards the practice of Medical Assistance in Dying, on identifying which core ideas may be underlying them, and on suggesting possible reasons for this disparity. The knowledge acquired contributes to understanding its complexity, shedding light into ambivalent profiles and creating strategies to increase their participation.

**Methods:**

We conducted an exploratory qualitative research study by means of semi-structured interviews (1 h) with 25 physicians and nurses from primary care (12), hospital care (7), and palliative care (6), 17 women and 8 men, recruited from Madrid, Catalonia, and Andalusia between March and May 2023. Interviews were recorded, transcribed, and coded in Atlas.ti software by means of thematic and interpretative methods to develop a conceptual model.

**Results:**

We identified four approaches to MAiD: Full Support (FS), Conditioned Support (CS), Conditioned Rejection (CR), and Full Rejection (FR). Full Support and Full Rejection fitted the traditional *for* and *against* positions on MAiD. Nevertheless, there was a gray area in between represented by conditioned profiles, whose participation cannot be predicted beforehand. The profiles were differentiated considering their different interpretations of four core ideas: end-of-life care, religion, professional duty/deontology, and patient autonomy. These ideas can intersect, which means that participants' positions are multicausal and complex. Divergences between profiles can be explained by different sources of moral authority used in their moral reasoning and their individualistic or relational approach to autonomy.

**Conclusions:**

There is ultimately no agreement but rather a coexistence of plural moral perspectives regarding MAiD among healthcare professionals. Comprehending which cases are especially difficult to evaluate or which aspects of the law are not easy to interpret will help in developing new strategies, clarifying the legal framework, or guiding moral reasoning and education with the aim of reducing unpredictable non-participations in MAID.

**Supplementary Information:**

The online version contains supplementary material available at 10.1186/s12910-024-01069-1.

## Background

Since the 1990s, and especially following its legalization in some countries, the provision of aid for dying has been progressively incorporated into clinical practice [1]. However, whether or not health professionals should participate in intentional bringing about patients’ death is a highly controversial topic. A tradition in which the preservation of life constitutes a supreme value persists in training and care work. According to that view, Medical Assistance in Dying (henceforth MAiD) is considered beyond and even contrary to professional duties [2–9]. Furthermore, deep personal convictions may make it difficult for professionals to help someone die without feeling that their moral integrity is damaged and some loss of identity [2–4, 6–8, 10–12]. Apart from these possible ethical conflicts related to moral conscience and intrinsic values, there may also be multiple contextual factors driving healthcare professionals to reject the practice of MAiD, including stigma, work overload, lack of institutional support, and fear of potential psychological impact, among others [13, 14].

The aforementioned personal, professional, and political circumstances may clash with a right to MAiD and patient autonomy to make decisions about the end of their lives.

In the case of Spain, the Organic Law 3/2021, regulating euthanasia and medically assisted suicide (henceforth LORE) was approved in 2021, and its implementation has been uneven throughout the territory. Unlike other countries in which there is no obligation for professionals to perform MAiD or refer the patient to another professional (e.g.The Netherlands, Belgium, Switzerland, to cite a few), the Spanish law declares that MAiD is a citizen’s right, like Canada [15]. Thus, the State has the obligation to ensure its access through the health services. This right to MAiD implies a series of duties for public administrations, including financing and guaranteeing it as a public benefit, but also duties for healthcare professionals working in both public and private healthcare institutions. These include the duty to provide adequate access to and care for MAiD (arts. 1 and 13 LORE), which some professionals may view as a contradiction to their moral convictions. Patients intending to request MAiD must be of legal age, residents in Spain, and: a) suffer from a serious, chronic, and incapacitating illness; or b) have a severe and incurable illness with a limited vital prognosis (art. 3 LORE). In both scenarios, proof must be provided of intolerable physical or psychological suffering that cannot be relieved. Patients also need to be competent at the time of requesting MAiD or have expressed their MAiD request in advanced directives.

A MAiD request involves input from two professionals (referring physician and consulting physician) and oversight to ensure all requirements are met by a *Commission of Guarantee and Evaluation* before the final acceptance. The LORE also includes provisions regarding the right to conscientious objection for all healthcare professionals directly involved in the MAiD process who believe that the duties derived from MAiD are incompatible with their convictions. The regulatory text states that the exercise of conscientious objection by healthcare professionals must not undermine equal access to MAiD quality of care (arts. 14 and 16 LORE), meaning that public administrations need to ensure that healthcare professionals exercise that right in a way that does not harm or hinder access to MAiD for those who request it. To facilitate that, the law requires objectors to disclose their conscientious objection by signing in registries where their confidentiality is protected.

According to the 2023 evaluation report on the provision of MAiD from the Spanish Ministry of Health [16], of the 576 MAiD requests submitted in 2022, 288 (50%) ended up in MAiD (236 by euthanasia, 11 by assisted suicide, 41 with non-specified data). This represents 0.064% of all annual deaths in the country. The 2022 report acknowledged that MAiD requests may be underreported in cases where the referring physician was against the practice or listed as a conscientious objector [16]. In some cases, delays have been attributed to health professionals’ undisclosed moral objections [17]. Although the report does not include specific data on this, qualitative research conducted with patients who have requested MAiD point to undisclosed conscientious objection as a major obstacle for patients to access MAiD [18].

Considering that the right to MAiD must be guaranteed, non-participations in MAiD may create access barriers and need to be explored and addressed. To understand the full range of ethical conflicts related to MAiD that healthcare professionals, as providers, may face, our research focused on two dimensions. On the one hand, we attempted an in depth understanding of conscientious objection and how it is defined, perceived and exercised by health professionals in Spain. On the other hand, we tried to identify and categorize the main ethical conflicts that are compromising healthcare professionals' participation in MAiD. This paper addresses the latter. It aims to refine the understanding of healthcare professionals’ positions when faced with MAiD requests, beyond the simplistic *for* and *against* dichotomy in all cases, and what core ideas may influence their moral judgments. We will discuss our findings applying the theoretical framework provided by the ethics of care and a feminist epistemology of moral reasoning. This research may contribute to understanding the complexity of the ethical dilemmas surrounding MAiD and highlight the different moral epistemologies that shape these conceptions. Furthermore, it may help to shed light on conditioned profiles, which require greater attention as their participation cannot be predicted beforehand. By doing so, this study may provide relevant insights for policy development on MAiD and how to acquire higher levels of healthcare professionals’ participation.

## Methods

This exploratory qualitative research was conducted by the members of the CONFINES^1^ research project, an interdisciplinary group of healthcare professionals, social scientists, and philosophers working in Spanish public universities, and with experience and training in interviews and qualitative methodology. We were guided by COREQ [19] in ensuring methodological and analytical rigor.

### Study design

The study was designed to be not just descriptive, but to create a conceptual model or explanation of the topic of analysis. As it includes sensitive aspects pertaining to the intimate sphere of individuals, we chose to use semi-structured interviews as the most appropriate technique to collect information. This method allows for a more fluid relationship between the interviewer and interviewee, a more private interview, and a more profound knowledge of the object of research than other techniques [20].

The interview script (see Supplementary File 1) was developed according to previous studies and parallel literature reviewed [14] and following the aims of the study. It was piloted with seven key informants, all of them healthcare professionals trained in bioethics, with vast experience in end-of-life practices, knowledge in implementing MAiD, and members of healthcare ethics committees or Guarantee and Evaluation Commissions. These interviews provided contextual information to approach the study objective and helped us to improve the final script. The present article is based on the results corresponding to Sects. 2 to 4 of the script (“Interviewee profile”, “Ethical conflicts at the end-of-life”, “Evaluation of the Spanish Organic Law regulating Euthanasia”).

### Participants and setting

We carried out the fieldwork with 25 healthcare professionals (physicians and nurses) from Andalusia, Catalonia, and Madrid between March and May 2023. We chose these three autonomous communities based on population size, case incidence, and the reject index of the practice [16, 17, 21, 22]. The main inclusion criterion was that they should be healthcare professionals potentially receiving MAiD requests in their workplace. We made an effort to include variability regarding different healthcare settings. We awarded primary care practitioners special consideration, as they are the ones who receive more MAiD requests (67% in Catalonia, and 92% in Andalusia, no data from Madrid). We also gave attention to hospital care (trying to include, when possible, specialists from different services involved according to the reports), and palliative care, as a particular service that serves as a link between hospital care and home care. The recruitment process attempted to reach balance in terms of profession (medicine/nursing), gender, and years of experience. Although we focused the research on the provision of MAiD in public healthcare facilities, which represents the 99% of all provisions, we also included some private centers with hospital or palliative care services, to explore possible differences (Table 1). Last but not least, we looked for a wide variety of pro- and anti-euthanasia profiles, as well as profiles with doubts, although this was not always possible to know prior to contact.

**Table 1:** Participants’ characteristics

Code	Autonomous Community	Gender	Profession	Healthcare setting	Public/Private	Specialty	Years of experience
A1_CR	Andalusia	F	Nurse	Hospital care	Public	Palliative	40
A2_FS	Andalusia	M	Nurse	Primary care	Public	Primary care	18
A3_FR	Andalusia	F	Doctor	Hospital care	Public	Palliative	5
A4_CR	Andalusia	F	Doctor	Primary care	Public	Primary care	7
A5_CS	Andalusia	F	Doctor	Primary care	Public	Primary care	31
A6_CS	Andalusia	M	Doctor	Hospital care	Public	Internal	8
A7_FS	Andalusia	M	Doctor	Hospital care	Public	Intensive	32
A8_FR	Andalusia	F	Doctor	Primary care	Public	Primary care	7
A9_FR	Andalusia	M	Doctor	Hospital care	Private	Internal	36
C1_FS	Catalonia	F	Doctor	Primary care	Public	Primary care	7
C2_FS	Catalonia	F	Doctor	Primary care	Public	Primary care	20
C3_FS	Catalonia	F	Doctor	Hospital care	Public	Neurology	18
C4_CR	Catalonia	F	Doctor	Primary care	Public	Primary care	31
C5_FR	Catalonia	F	Nurse	Primary care	Public	Primary care	20
C6_CR	Catalonia	F	Nurse	Hospital care / Primary care	Public	Palliative	10
C7_FS	Catalonia	F	Nurse	Primary care	Public	Primary care	23
C8_FS	Catalonia	F	Nurse	Primary care	Public	Primary care	6
M1_FR	Madrid	M	Doctor	Hospital care	Private	Palliative	30
M2_FS	Madrid	F	Doctor	Hospital care	Public	Neurology	12
M3_CS	Madrid	M	Doctor	Hospital care	Public	Neurology	8
M4_FS	Madrid	F	Doctor	Primary care	Public	Palliative	30
M5_CS	Madrid	F	Doctor	Primary care	Public	Primary care	6
M6_FS	Madrid	M	Nurse	Primary care	Public	Primary care	30
M7_FR	Madrid	M	Doctor	Hospital care	Public	Palliative	20
M8_CR	Madrid	F	Nurse	Nursing home	Private	Geriatrics	25

We followed different sampling selection strategies to ensure the broadest and most diverse representation. On the one hand, purposive and intentional sampling. We looked for any informal contacts the research team might have (some were healthcare professionals, others individuals in contact with healthcare professionals due to their teaching and research roles). On the other hand, we used snowball sampling. To be respectful of all professionals’ intimate perspectives on MAiD, we asked the interviewees if they knew someone who could meet the criteria defined by the sample design. If a potential participant wanted to know more about the project, we sent her an information sheet; researchers did not report personal reasons for their interest in the research topic to the interviewees. In case of acceptance, a research team member made the contact by phone and arranged an interview. There was no direct relationship with participants established prior to the interviews.

### Data collection

The interviews lasted around an hour, and were collected between March and May 2023. During each interview, the script was adopted as an open guide that could be slightly modified depending on the context and profile of the interviewee. We offered participants the option of doing the interview in person or online/by phone. We found no substantial differences between the two methods in terms of how the interviews were carried out. Data was collected either in their workspace or at home, with no other presence except for the participant and the researcher.

The healthcare professionals received information about the study and agreed to participate voluntarily after signing an informed consent form, which stated that the interview was anonymous but would be recorded for later analysis. They had the opportunity to ask any questions or raise any doubts they might have about the study before the beginning of the interview and did not receive any financial reward for their participation in the study. Due to the sensitive topics addressed during the interviews, the research team put much effort and care into emphasizing all aspects of privacy and data management, ensuring that only members of the research project could access this sensitive information. The interviews were audio-recorded and transcribed. During the data collection the research team met to discuss data saturation and the need to include/exclude some participants based on whether their profiles were already covered or not.

### Data analysis

Following Neem et al. [23] conceptual model in qualitative research, we describe the process of thematic analysis leading to four professional profiles and the underlying core ideas. First, transcription and familiarization with the data. Second, selection of keywords. Third, coding. A multidisciplinary team analyzed and encoded the transcriptions thematically using Atlas.ti, a qualitative data software. This method has proven to be “a robust, systematic framework for coding qualitative data, and for then using that coding to identify patterns across the dataset in relation to the research question” ([24], p. 1–2). We created a coding tree based on the research questions and the field findings, combining closed categories with emerging ones. In case of disagreement between coders, the coding team met and discussed the relevance and adequacy of the categories. The coding tree was piloted in the first interviews and subjected to changes to improve its effectiveness and scope. The final version of the coding tree had 44 codes, plus an emerging open code to collect contributions that were relevant to the study but that could not be included in other codes. Fourth, theme development (we merged codes into eight themes: context, barriers to the application of MAiD, actions at the end of life, doubts, motives, LORE, opinions about conscientious objection, and proposals). Fifth, conceptualization through interpretation of keywords, codes and themes. We identified four main core ideas that allowed the team to interpret and structure the results (*end-of-life care* [options, experiences, impact, doubts, context], *religion* [religious motives, doubts], *professional duty* [duty, deontological motives] and *patient autonomy* [options, experiences, doubts, opinion]). Sixth, development of a conceptual model. According to the discourses regarding the four core ideas, we conducted an interpretative analysis to divide healthcare professionals interviewed into four profiles/groups according to their positions and actions: Full Support, Conditioned Support, Conditioned Rejection, and Full Rejection to Medical Assistance in Dying. These profiles are detailed in the Results section.

## Results

We discerned a variety of participants’ opinions and practices related to MAiD and end-of-life care. Although there was overall consensus on some issues, like the general duty of healthcare professionals to accompany their patients at the end of their lives, there was greater disagreement among others, such as understanding professional responsibility or patient autonomy and how, when, or in which cases it is morally acceptable to perform MAiD.

**Fig. 1:**
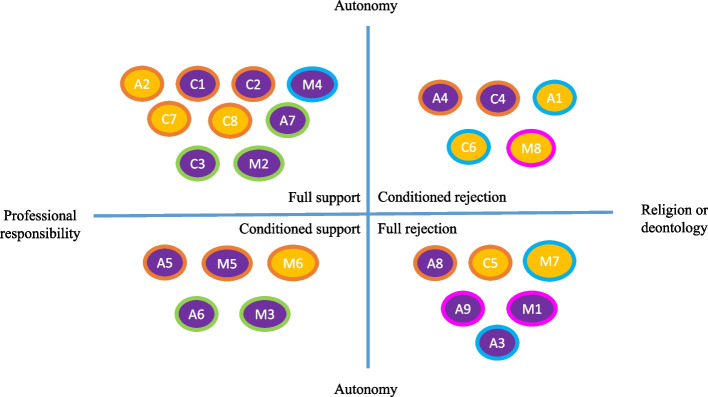
Discursive profiles around MAiD. Internal circle colors represent physicians (purple) and nurses (orange). External circle colors represent the specialty: primary care (orange), palliative care (blue), hospital care (green), and private institutions (pink)

To analyze this plurality of approaches, we created four analytical types of discourse (see Fig. 1): Full Support (FS), Conditioned Support (CS), Conditioned Rejection (CR), and Full Rejection (FR).

FS and FR profiles included the in favor/against positions to MAID and their active participation or non-participation in the practice under all circumstances, with no exceptions. FS participants argued that provision of MAiD is a citizen's right and therefore a responsibility of healthcare professionals. In contrast, whether for religious reasons or based on a deontological argumentation, FR participants stated their belief that euthanasia is a boundary that should not be crossed, at least via medical practice. In this respect, they tended to argue that there is a moral difference between causing death and just letting die, the former entailing a burden they do not wish to bear.

Between these two opposing positions we found a group of ambivalent profiles, whose discourses can overlap and whose practices may not necessarily differ. The main reason for distinguishing them is that CS participants are in favor of MAiD and would generally participate in all the steps of the process but they are conditioned by the case context and circumstances whereas CR participants are in general against MAiD, but could exceptionally accept to participate, generally with restrictions to perform the drug injection.

In the following sections we provide more detail on these profiles by showing how they relate to four underlying core ideas: End-of-life care, religion, professional duty, and patient autonomy.

### Main core ideas shaping different healthcare professionals’ profiles with regard to MAID

#### End-of-life care

Regarding familiarity and comfort towards end-of-life care, most interviewees (with independence of their profile) perceived a taboo around death in healthcare environments and a lack of training and reflection on end-of-life approaches and accompaniment in most healthcare education programs. This general perception, according to them, can sometimes lead to death avoidance, as many healthcare professionals do not consider requests for end-of-life accompaniment to be aimed at them directly.

Health professionals do not want to see a dying person even in the slightest. (A9_FR).

[There are health professionals] who take a low profile. (M4_FS).

Some participants mentioned that physicians are usually trained to save lives and see death as a failure of their efforts:

*The feeling of failure often goes hand in hand with patients dying. [...] Keep trying no matter what brings a feeling of failure and an ethical conflict for physicians.* (A3_FR)

There was a broad consensus on the importance of end-of-life care in all its forms among the personal experience and values of those interviewed. Several of them, regardless of their profile, pointed out that accompanying patients at the end of life is among the most powerful experiences of their professional careers. These can be intense but rewarding experiences, in which professionals might feel a unique intimacy and commitment.

*I have always handled the topic of end of life well and have always liked it. I have had my best and most beautiful experiences with people close to death, with people who were already at the end of their lives.* (A5_CS)

Nevertheless, we identified divergences on what each of the groups considered to be end-of-life care, and how compatible or incompatible different practices could be. These divergences should be understood as a scale gradation more than pure discernible compartments. On the one side of the spectrum, FS profile understands that MAiD is a logical continuity of end-of-life care, just as palliative care is. According to them, palliative care is a clinical decision made by healthcare professionals, while MAID is a personal decision based on the patient’s perception of her life. These different practices can be used by different kinds of patients with different needs.^2^

*Palliative care professionals visit patients that need palliative care, and they offer them one thing. Patients can need MAID, and that’s another thing, even if it is the same patient in different periods of her life. There is no confrontation between both.* (A7_FS)

CS participants recognise MAiD as part of end-of-life care options, but they have some doubts of its convenience in certain circumstances. In other words, they might have a more restrictive approach than the current access conditions recognised by law. The recognition of MAiD as a suitable form of end-of-life care is mediated by a time factor, for example. It is perceived as more challenging when it comes to patients with no foreseeable death in the near future, where it is more difficult to be sure about an irreversible prognosis (e.g., requests due to neurodegenerative diseases in their intermediate states with no current cure, although some new treatment may be discovered in the future).

*[I] would prefer the disease to be in a late stage, not at the early stages*. *[...] If you have a limitation and you know that it will increase, I would face it better if you request it at a late stage. (M5_CS)*

They can also need extra safeguards to feel sure before accepting a MAiD request, like a deep exploration on the casuistry, the patient’s biography or the social context of the request.

*I believe that it depends on the case, the situation, everything.* (A5_CS)

FS participants also acknowledge the complexity of the context in which the MAiD is requested but it does not seem to create as many concerns about the moral result of their decision.

On the other side of the spectrum, FR participants consider that end-of-life care does not include the option of hastening a death, and thus, MAiD. Sometimes the grounds to justify their view are related to the core idea of deontology and professional values. Some others, it is connected to the core idea of religion and the sanctity of life, as we will see in the next sections.

Only two of the six interviewees of FR group mentioned palliative care in contrast to MAiD. According to them, palliative care is considered the expertise area of suffering and, thus, should be the solution for these unbearable suffering that patients report.

*Very few patients, terminal patients, palliative patients, that could request MAiD due to vital suffering would choose that option if they were offered a palliative sedation instead.* (A3_FR)

*We have to try everything before the patient has a MAiD, in the same way that we try everything before someone commits suicide. In this sense, for me it is necessary that all MAiD requesters be visited by a palliative care specialist.* (M1_FR)

Finally, CR participants are usually aligned with FR discourses, and do not understand MAiD as an end-of-life care option. They defend the need to accept the fragility and vulnerability of human life, as well as the need to make peace with the disease and foreseeable death. Nevertheless, they leave a door open for exceptional cases (e.g., when the requests come from patients in a very late stage of their disease and under great suffering).

*I understand that there might be some cases, but very isolated, that need MAiD, but those would be very exceptional cases, not as ordinary as the law considers.* (M8_CR)

#### Religion

All participants who identified themselves as religious followed the Catholic faith. The four profiles identified religion as the main common and genuine reason for being against MAiD among healthcare professionals. Nevertheless, there was a plurality of approaches regarding the potential accommodation between MAiD and religious beliefs, and not all of them ended in a negative of participation. These results show a much more complex and rich conception of this core idea.

When religious beliefs are mentioned, FR profile feels an insuperable moral contradiction. Life is something sacred, given by a supreme being, and, thus, humans should not interfere with God’s will.

*Life is sacred from conception, when the ovule and the spermatozoon come together, until the natural death of the individual. [...] We are unique projects of God.* (C5_FR)

According to these professionals, MAiD devalues life, and taking a life through such a procedure makes them feel very morally uncomfortable. However, religion was not the most brought up core idea to justify FR positions.

CR participants who are religious and only in favor of MAiD as an exceptional practice sometimes seem to give more importance to other values within the Catholic faith. This view can make them opt for MAiD, without collaborating in the final drug injection. They report a strong sense of commitment and loyalty towards the patient.

*The commitment and the bond with the patient are strong for me. And if he decides that I should be there, I would be failing him if I wasn’t.* (C4_CR)

Although commitment and loyalty can also be interpreted in terms of professional values, and not religious ones, we find similar quotes in religious CS participants that endorse the religious interpretation. A5_CS expresses something similar when declaring the following:

*That person trusted his doctor and believed in his support and accompaniment. And maybe I should be there to…make it all more natural [...] It must be a beautiful act, a compassionate act, merciful*.

Mercy, compassion, loyalty, or helping the neighbor can be the religious basis that allow a conditioned positive view of MAiD.

#### Professional duty and deontology

One of the main discussions that had an impact on all discursive profiles is whether MAiD is part of a healthcare professional’s duties or not. The current deontological code for medicine from the Collegiate Medical Organization of Spain says that physicians shall not intentionally provoke or assist in a patient's death (Art. 38.4). However, in the final dispositions, it specifies that “a physician acting under the protection of the laws of the State cannot be sanctioned deontologically” [27]. This last version, which is aligned with the World Medical Association Code of Ethics [28], was updated in December 2022, several months after the approval of the LORE.

The use of deontological arguments to defend one’s perspective on MAiD varied significantly among the interviewees, but there was general consensus that a contradiction exists between the current code and the practice of MAiD. Some interpretations of the professional deontological code (both in nursing and medicine) elevate it to a principle of mandatory compliance in all cases, as if it were a legal principle. Some may consider the situation from a position of casuistry, leading them to question the universality of the deontological code in case of suffering and no other means to end it. Still, others consider it just a list of recommendations, and the leading professional obligation is to fulfill a citizen’s right recognized by law.

Positions against MAiD frequently base their arguments on the idea that either MAiD is not coherent with the deontological code or it collides with healthcare professions principles. For the most part, FR participants claim that the code explicitly states that death should not be induced, and that MAiD cannot therefore be performed.

*A doctor cannot kill; under any circumstance, not even if a patient explicitly requests it. That is what the Deontological Code says.* (M1_FR )

It is also understood as a maleficence towards the patient.

*I do not consider MAiD a treatment that benefits them, I would not use nor this nor any other option that I consider that it would harm them*. (A3_FR)

These arguments are common ground for some CR participants too.

*It is outside my values, which are part of my profession, which is the one I chose.* (A1_CR)

Sometimes, the internal logic behind these deontological reasoning is not against the right to the provision of MAiD itself, but against it being carried out by healthcare professionals. Hence, we can find claims that MAiD should not be a medical or a healthcare act.

*Perhaps we should create another figure outside the healthcare system, like another public agency in charge of suicides.* (A3_FR)

Yet, as previously seen, CR participants might also exceptionally accept the convenience of MAiD when no other option is available and might focus more on other professional values. Their commitment to the patient is an ethical value of the healthcare profession that might be put ahead of other deontological arguments. Some express it in terms of a trust relationship based on years of monitoring.

*What made me participate in MAiD was the patient, the confidence that he had in me staying with him during the whole procedure.* (A4_CR)

*Because I know you, I know that there is no other way, I love you, and I know that that’s the option that you want, because truly I don’t have any other resource to offer you.* (A1_CR)

At large, they do not understand MAiD as a harm for the patient, but as an extraordinary option to avoid suffering, which allows them to interpret the deontological code in other terms.

CS participants also took trust and commitment to the patients’ needs as important values of their healthcare professionals.

*If it were me, the one requesting MAiD, I would want my GP to be there for me, because he would know me well.* (M5_CS).

They usually pointed out that MAiD is a logical extension of end-of-life care that brings some peace and safe spaces to patients and families, since they can choose where and in the presence of whom they want to die.

Finally, FS participants understood MAiD in terms of aiding instead of killing. As C8_FS argued, “*If euthanasia is anything, it is the intention to die well*”. The underlying reason for that is a comprehension of professional values as a form to accompany, alleviate suffering when there is no cure, and comfort patients.

*I am a nurse by calling, and I like to help, from birth to grave. [...] I am not killing a patient, I am helping him to have a decent death.* (A2_FS)

Furthermore, MAiD is an option recognized by a democratic state law, a benefit that healthcare professionals have to provide, which is a right of the patients. Thus, FR participants defend that providing MAiD “*puts you in a position of responsibility towards the other*”. (C3_FS).

*There is a law, a patient’s right … that we need to offer. It is a responsibility.* (A2_FS)

#### Patient autonomy

Other topics that range the four discursive profiles are the degree of recognition given to patients’ autonomy, the respect healthcare professionals should have for their patients’ decisions, and whether there is an obligation to act according to them.

There was a general consensus on the importance of the first two, but disagreement on the latter. Healthcare professionals who advocate for MAiD (FS and CS) place a greater emphasis on the first factor. They expressly point out that the request is just the last step of a difficult process and not a whim. They also acknowledge that these decisions are generally reasoned and matured long before they reach the formal request to the practitioner, and taken very seriously. These desires are sustained over time and transmitted clearly and openly after having weighed-up alternatives. Once proven that they meet the criteria established by law, it is not a matter to judge what would you do in the same situation or what do you consider best, but what do the patient want, as owner of her life. There is a need to understand:

*How the patient feels, the desire, regardless of whether you say: ‘well, maybe it is not bad enough to do it right now... maybe in two, three years, depending on how he progresses…’ But, let's be honest, what the patient does not want is for the disease to progress for two or three years until she can no longer do anything.* (C1_FS)

CS participants will respect patients’ reasoned decisions and their sincere will to bring death forward. A6_CS notes that *every person has the autonomy to decide how to live and in which conditions*. However, as mentioned in *End-of-life care* previous section, they perform MAID on a case-by-case basis, and they might have doubts about the appropriateness of the decision, especially in those cases where there is a slow and progressive deterioration. Patients’ autonomy, then, seem to be nuanced by extra safeguards (e.g., informal talks with colleagues, revision of existing literature, debates on prognosis uncertainty, reassurance of other possible social or economic needs not covered, and so on). A6_CS needed to *Study the case very well* and make sure that *the patient really knows what he is asking for, what he needs*.

We can find similar doubts on some CR participants. They respect patients’ reflected decisions but they have epistemic and moral dilemmas that change their predisposition to act in accordance to patients’ wills, and it may even be perceived as demands.

‘*I demand MAiD, it is my right!’ The patient expressed it like this: ‘I demand this and I have an incapacitating disease that creates suffering…’ and suffering is something so difficult to quantify. I have to believe you. Even if you are saying it with a smile on your face, I can’t contradict you.* (A1_CR)

Finally, FR participants also claimed that they respected patients, but were unwilling to comply with their wishes. Some advocated patients’ autonomy to do what they want as long as they do not ask other people to fulfill their wills.

*Well, I would respect your decision, that is to say, I would not oppose it; I mean, my attitude would be one of respect. Having said that, what you cannot expect from me is to carry it out. That's it.* (A9_FR)

Some others were dubious about patients’ autonomy by reasoning that they do not always know what they mean.

*They can experience such moments during different stages of the disease. At that moment, what they express is the desire ‘I would like to be dead.’ And sometimes the way to express that wish is saying ‘euthanasia,’ even if they don't specifically want euthanasia, or they don't understand what it means.* (M7_FR)

Although all profiles made sure that the patient had all the information before requesting MAiD, FR participants were much more concerned about possible misconceptions or lack of knowledge or capacity to discern what they want. We will dive more into it in the discussion section.

## Discussion

This study reveals that traditional categorical positions in favor of (FS) and against (FR) MAiD under all circumstances persist among Spanish healthcare professionals following the approval of the LORE. These results were expected and aligned with literature debates on the topic [2, 4, 6, 7, 29, 30]. Such studies report, on the one hand, a general recognition of patient autonomy, and their right to ask for MAiD and, on the other, great discrepancy between healthcare professionals who find their values and professional commitment challenged by requests for MAiD, and those who believe that refusing MAiD is a greater cause of harm for their patients.

A novel discovery is the identification and description of two conditioned profiles (CS and CR), whose specific position on MAiD can change under certain conditions. Sometimes, their reasons for accepting or refusing to participate in a MAiD request are grounded in an ethical conflict that can be triggered by one specific case but not another. The variability in their reactions is contemplated by the legal protection of conscientious objection, which can be claimed and unclaimed as many times as necessary. These variations pose a challenge for public institutions in charge of MAiD provision and their prevailing interest of preserving patients’ right to MAiD.

The elaboration of four core ideas underlying these four profiles (end-of-life care, religion, professional duty, and patients’ autonomy) is in line with results of previous qualitative research in which discursive keys such as professional identity, commitment to patient autonomy, and personal values and beliefs had emerged [31]. Nevertheless, none of these ideas alone is sufficient to explain healthcare professionals' positions. On the contrary, it shows how their views are based on a complex interaction between these notions. For instance, FR and CR participants meet at an intersection between religious and deontological reasons, although they devote more arguments to the second one when defending their position. Findings in other studies also report that only a minority of clinicians explicitly identified religious values as relevant to their position against MAiD [6, 32–34]. If we consider, for example, the notion of commitment with the patient, it can be justified in terms of autonomy, professional duty, or religious values.

Therefore, professionals’ standpoints are usually multifactorial. We can find the importance of a long-lasting relationship, respect for patients’ autonomy, no other end-of-life care options, and commitment (that can be understood in deontological or religious terms) all in one argument (see A1_CR in the results section, p. 18, lines 410 to 414).

Differences in the results of each core idea can be explained on the basis of whether profiles use an external moral authority to be followed under all circumstances or an internal moral authority applicable on a case-by-case basis, as discussed below.

Of the four core ideas, we will focus on *professional duty* and *patient autonomy* due to their level of both epistemological and metaethical disagreement. The participants’ positions clashed because they have different understandings and definitions of these topics. To defend this hypothesis, we will use the theoretical frameworks of care ethics [35, 36] and a feminist epistemology of moral reasoning [37–39].

### Sources of moral authority—Deontology and professional values

Healthcare professionals’ positions are built on different assumptions about the sources of moral authority and their purpose. In the case of deontology and the deontological code, there is a set of principles and values that guide each profession.

We can consider the deontological code as a compact set of law-like prepositions that prescribes the moral behavior of a rational moral agent, an individual action-guided system. Then, we assume that morality can be codified in a core of universal values equally valid as a guidance system for all moral agents in all moral circumstances, with independence of any time, clinical context, or differences between societies or agents’ positions [39]. Nevertheless, feminist epistemology of morality and care ethics have stressed that no moral model is actually independent of the social context in which it is created [39, 40]; claiming this independence is just a socially built way of creating and validating moral authority by a ruling powerful minority [39].

From this perspective, we can consider the deontological code as an agreement of what is socially understood as morally correct, and admit that it is open to interpretation. This approach depicts morality as a continuing negotiation among people, “a practice of mutually allotting, assuming, or defining responsibilities of important kinds, and understanding the implications of doing so.” ([39], p.67).

This underlying comprehension of the basis of morality is connected to the four profiles. FR discourses can be an example of the first, non-contextual model of morality. Their rejection of the practice is based on awarding a universal external authority to the deontological code, even against applicable legislation on MAiD (LORE).Their arguments are rooted in the Hippocratic tradition of life preservation. MAID is considered to be a harm, and it goes against the principle of non-maleficence.^3^ According to them, the deontological code explicitly says that healthcare professionals cannot provoke a patient’s death.

Indeed, MAID can be perceived as a challenge to professional values, as reported by different profiles. Other studies from Belgium, Switzerland, and the Netherlands report similar results: clinicians might find their professional identity and duties in confrontation with MAiD [6, 7, 33].

For other profiles, there seems to be more negotiation of how to balance different professional values and how healthcare professionals can interpret them or prioritize one over another. Positions in favor of MAID or with certain doubts might focus on other professional values.

In FS discourses, there seems to be no moral dilemma, since the law already reflects their position with regard to MAiD. Therefore, they defend their professional participation as part of a task guided by the regulations in force. The deontological code is not considered the primary source of moral authority, and they prioritize other moral values as responsibility, avoiding suffering, and accompanying a good death when cure is not possible.

The more moderate or ambivalent profiles (CS and CR) show a greater use of internal sources of moral reasoning. Rather than giving full authority to the deontological code or the law, their own moral reasoning plays a crucial role in every case they come up against. They tend to embrace the chance of different outcomes when facing a moral dilemma. They accept that this variability depends on the context, or even the possibility to change their own moral position through time. Nevertheless, as we have seen in the results section, their initial positions differ. Patel et al. distinguish between supporting MAID and not obstructing MAID [31]. This differentiation can allow us to explain CS and CR profiles. CS participants support MAID and have a similar discourse to FS participants regarding the most important professional values. In case of doubt, they will focus on avoiding suffering and providing a good death when there is no cure or accompanying the patient. CR participants are unwilling to participate and prefer other options, but find alternative ways of not obstructing the patient’s will according to their professional values (like the duty of non-abandonment, and the commitment to the patient).

### Different models of autonomy: Individualistic autonomy or relational autonomy?

We can define patient autonomy as a patient’s right and capacity to make decisions that affect her health status without being influenced or disregarded by healthcare professionals, family members or religious leaders. According to the results, all four profiles seem to recognize the value of patients’ autonomy, but there is no unitary agreement on how healthcare professionals should act in front of a patient's autonomous decisions in MAiD. This can be explained by the different notions of autonomy at stake, which have been conceptualized from different frameworks [40].

Respecting patient autonomy may collide with the FR perspective on what is a good practice for end-of-life care, as shown in other studies [32, 41]. Participants respond in two ways when confronted with patients' autonomy. On the one hand, they might question patients’ cognitive capacity to understand and choose what is best for themselves, or undervalue patients’ experience and preferences. On the other hand, they may recognize patients’ autonomy, but do not feel compelled to act according to it. They argue that patient autonomy cannot be an imposition over their professional duties, and frame the ethical conflict in terms of opposing individual rights: the patient’s right to access MAiD, and the professional’s right to not provide it, appealing to conscientious objection.

Both responses can be understood as an individualistic perspective on autonomy based on the liberal tradition of the self (independent, individualized decision-making by isolatable rational individuals). Under this model, moral agents understand the moral conflict as an opposition between individual rights of rational actors, based on universal principles to be applied under all circumstances, and with no social context involved. In their systematic review, Gómez Vírseda et al. [40] stated that apart from being a nonrealistic conception of the self, the literature also highlights that it is an inadequate portrait of decision-making, and one that does not incorporate social reality. Furthermore, it is criticized because it includes discriminatory prejudice, and represents a shortcoming in current practices, laws, and policies.

Feminist scholars have introduced another perspective on autonomy called relational autonomy [37, 38] based on a different definition of the self [35, 37]. To them, we are relational selves in social relations that have an impact on us. When faced with a moral dilemma, the relational self gives importance to the context, to particularism, and to the responsibilities towards the other, instead of framing the conflict in terms of competing rights based on universal principles [36]. From this perspective, relational values, such as compassion, empathy, and responsiveness, should be considered in the decision making process [38, 40].

CR, FS, and CS profiles might, therefore, display a more relational conception of autonomy, considering their patients’ wills inserted in a network of needs, preferences, values, and relationships that they need to watch for.

Although the CR profile shares some of the FR characteristics, in exceptional cases their approach can include some degree of relationality. Thus, they can be more open to considering patients' wishes and narratives.

FS participants usually argue that patients’ wishes and autonomy should prevail as long as these are aligned with the parameters established by current law, something also mentioned in other studies [6, 42]. They emphasize patients’ experience, context, and cognitive competence.

CS participants work on a case-by-case basis, and thus, patients’ autonomy can be nuanced by extra safeguards related to an ethical and epistemic problem: uncertainty [43]. In some cases, they can be less sure about how to interpret the criteria established by legislation (e.g., ‘unbearable suffering’). The lack of specificity on MAiD regulations requires individual interpretation, which can be difficult, according to studies from multiple countries [6, 32, 42, 44–47]. This situation leaves room for the incorporation of personal perceptions, values, and understandings of the criteria that can be more restrictive than the law and disturbing for health professionals, as our results show. Therefore, CS participants might need some exchange of impressions from their colleagues that confirms patients’ narrative, diagnosis, and the suitability of the request. Moreover, uncertainty is also epistemic: In light of some health conditions with a descriptive prognosis, they fear a possible range of errors in predicting foreseeable death (e.g., requests due to neurodegenerative diseases in their intermediate states with no current cure, although some new treatment may be discovered in the future). In such cases, patient autonomy can be undervalued in the professional’s search for “objectivity” in decision-making processes. These findings were consistent across countries with older and newer legislation, demonstrating the need for healthcare professionals to learn and adapt to providing MAiD [4, 6–8, 30–34, 42, 46, 47].

### Strengths and limitations

These significant findings have been made possible thanks to the use of a qualitative methodology, which allows for a more in-depth and rich analysis of professionals’ responses than other methods. The study provides descriptive information on hitherto unknown results and gives a philosophical analysis to interpret them. Nevertheless, it does have some limitations. Although the results are promising and fill a gap in the current knowledge, they can only be extended to parts of the country (i.e., urban areas are more represented than rural ones). The use of convenient samples has the limitation that findings cannot be easily generalized to other populations [48]. It would therefore be interesting to complement these results with a quantitative study. We conducted the study in 3 of the 17 Spanish autonomous communities. A national study of all autonomous regions would therefore be interesting in order to be able to compare results.

One-third of the professionals we interviewed belonged to the conditioned profiles, so it is essential to keep analyzing which destabilizing factors or topics may generate ethical dilemmas (e.g., type of suffering, treatment alternatives, type of relationship with patient, mental health, or other forms of social support) and lead them to reject their participation in MAiD. Searching for common trigger cases and conflicts that can arise would help address areas that require more debate, consensus, and guidelines on how to act, avoiding certain obstacles regarding patients’ access to MAiD.

Finally, most of the interviewees were professionals from the public healthcare system (which provides 99% of MAiDs). However, there could be noteworthy differences between the public and private contexts, especially considering that most of Spain's private and semi-private healthcare institutions are religiously oriented. Further research is required to glean a more in-depth knowledge of professionals’ discourses and the potential influence of their workplace on their attitudes and practices. There is an urgent need to debate the different models of morality currently in existence.

## Conclusions

Healthcare professionals display a variety of positions regarding MAiD. We found four analytical discourse profiles that affect professionals’ participation in the practice: Full Support (FS), Conditioned Support (CS), Conditioned Rejection (CR), and Full Rejection (FR). These profiles are determined by different interpretations of or a lack of consensus on four core ideas: End-of-life care, religion, professional duty and deontology, and patient autonomy. These ideas can intersect, which means that participants' positions on MAiD are multicausal and complex. One way to explain these results is by considering care ethics and feminist epistemology of morality to distinguish which moral models underlie each position.

On the one hand, we find an external authority (legal or deontological) that must be applied in all cases. FS participants follow a legal external authority. They face no moral dilemma because the law already corresponds to their moral position. They understand that patients’ wishes should be fulfilled, and that it is their responsibility, part of the continuum of care. In contrast, FR participants follow a deontological moral authority, and claim that hastening death goes against the deontological code. The code is considered a universal guidance system for all moral agents in all moral circumstances, with independence of time, clinical context, or differences between societies. According to this view, they may recognize patients’ autonomy, but do not feel compelled to act according to it. They understand this moral conflict in terms of an opposition between individual rights.

On the other hand, the more moderate or ambivalent profiles (CS and CR) show a greater use of internal sources of moral reasoning. Rather than giving full authority to the deontological code or the law, their own moral reasoning plays a crucial role in every case they come up against. They tend to embrace the chance of different outcomes when facing a moral dilemma due to particular and contextual factors and case-by-case decisions. Thus, their active participation in MAiD cannot be predicted. CR, FS, and CS profiles might display a more relational conception of autonomy, considering their patients’ wills inserted in a network of needs, preferences, values, and relationships that they need to watch out for. Patient autonomy is essential to the CS profile, but it is sometimes nuanced by extra safeguards as they might have a more restrictive interpretation of the criteria established by law. In the case of CR, when facing a long-lasting relationship with a patient and no alternatives left to alleviate suffering, they will also consider exceptional participation in MAiD.

All the aforementioned show that there is ultimately no agreement but rather a coexistence of plural moral perspectives regarding MAiD among healthcare professionals. Instead of focusing only on the traditional and categorical *for* and *against* positions, there is a need to work on conditioned profiles, which can play a key role in the implementation of the law in Spain. Comprehending which cases are especially difficult to evaluate, or which aspects of the law are not easy to interpret and lead to uncertainty, will help in developing new strategies, creating supplementary assessment for healthcare professionals, clarifying the legal framework, or guiding moral reasoning and education. Not addressing these topics would result in unpredictable non-participations in MAID and, thus, barriers to a patients’ right that the State must guarantee and provide. Finally, we advocate for ethical and policy frameworks to embody a more relational perspective, since it can contribute to a better moral understanding between healthcare professionals and patients.

## Supplementary Information

Supplementary Material 1.

## Data Availability

The datasets generated and/or analyzed during the current study are not publicly available to preserve participants’ privacy, but are available from the corresponding author on reasonable request.

## Abbreviations

MAiD: Medical Assistance in Dying
LORE: Ley Orgánica Reguladora de la Eutanasia [Organic Law Regulating Euthanasia]
FR: Full Rejection to Medical Assistance in Dying
CR: Conditioned Rejection to Medical Assistance in Dying
CS: Conditioned Support to Medical Assistance in Dying
FS: Full Support to Medical Assistance in Dying

## Footnotes

1 It is aimed to analyze ethical conflicts and conscientious objection at the end-of-life practices, specifically regarding euthanasia and medically assisted suicide. More information at: https://www.inedyto.com/confines.html.

2 Although the Spanish Official Annual Report does not include information on the number of patients requesting MAiD that were also in palliative care programs (unlike the Canadian Official Annual report, for example, see reference [25]), the Annual Report from the Guarantee and Evaluation Commission of Catalonia, the Autonomous Community with more MAiD requests in Spain, states that people requesting MAiD were always offered palliative care, “nevertheless, although they could access palliative care, all of them expressed that the suffering derived from the underlying disease and their current vital situation were something they could not bear anymore” ([26], p. 19). The report also mentions that all those cases who could not complete the request in time due to a rapid deterioration of the disease received comfort measures and symptomatic control through palliative sedation ([26], p. 22). Unfortunately the Andalusian report does not include data on this topic and Madrid does not even have a public report.

3 A Swiss study reported that some other professionals might understand it the other way around: refusing MAiD as a greater cause of harm [6].

## References

[1] Boorse C. Goals of medicine. In: Giroux E, editor. Naturalism in the Philosophy of Health. History, Philosophy and Theory of the Life Sciences. Switzerland: Springer Cham; 2016. p. 145–77.

[2] Carpenter T, Vivas L. Ethical arguments against coercing provider participation in MAiD (medical assistance in dying) in Ontario Canada. BMC Med Ethics. 2020;21:46. 10.1186/s12910-020-00486-2. 32493374 10.1186/s12910-020-00486-2 PMC7271423

[3] Emanuel EJ, Onwuteaka-Philipsen BD, Urwin JW, Cohen J. Attitudes and practices of euthanasia and physician-assisted suicide in the United States, Canada, and Europe. JAMA. 2016. 10.1001/jama.2016.8499. 10.1001/jama.2016.8499 27380345

[4] Fontalis A, Prousali E, Kulkarni K. Euthanasia and assisted dying: What is the current position and what are the key arguments informing the debate? J R Soc Med. 2018;111:407–13. 10.1177/0141076818803452. 30427291 10.1177/0141076818803452 PMC6243437

[5] Organización Médica Colegial de España-Consejo General de Colegios Oficiales de Médicos. El CGCOM ante la aprobación del Congreso de los Diputados del dictamen de la Comisión de Justicia sobre la Proposición de Ley Orgánica para la regulación de la eutanasia en España [The CGCOM, in view of the approval by the Congress of Deputies of the opinion of the Justice Commission on the Proposed Organic Law for the regulation of euthanasia in Spain]. 2020. https://www.cgcom.es/notas-de-prensa/el-cgcom-ante-la-aprobacion-del-congreso-de-los-diputados-del-dictamen-de-la. Accessed 6 May 2024.

[6] Otte IC, Jung C, Elger B, et al. “We need to talk!” barriers to GPs’ communication about the option of physician-assisted suicide and their ethical implications: results from a qualitative study. Med Health Care Philos. 2017;20:249–56. 10.1007/s11019-016-9744-z. 27785588 10.1007/s11019-016-9744-z PMC5487738

[7] Snijdewind MC, van Tol DG, Onwuteaka-Philipsen BD, et al. Developments in the practice of physician-assisted dying: perceptions of physicians who had experience with complex cases. J Med Ethics. 2018;44:292–6. 10.1136/medethics-2016-103405. 27495234 10.1136/medethics-2016-103405

[8] Sprung CL, Somerville MA, Radbruch L, et al. Physician-assisted suicide and euthanasia: emerging issues from a global perspective. J Palliat Care. 2016;33:197–203. 10.1177/0825859718777325. 10.1177/0825859718777325 29852810

[9] World Medical Association. Declaration on Euthanasia and Physician-Assisted Suicide. 2019. https://www.wma.net/policies-post/declaration-on-euthanasia-and-physician-assisted-suicide/. Accessed 6 May 2024.

[10] Fernandez-Lynch H. Conflicts of Consciencein Health Care: An Institutional Compromise. Cambridge Mass: The MIT Press; 2008.

[11] El T-C. peso de la conciencia: la objeción en el ejercicio de las profesiones sanitarias [The weight of conscience: objection in the practice of health professions]. Madrid: CSIC; 2014.

[12] Wicclair MR. Conscientious objection in health care: an ethical analysis. Cambridge: Mass. Cambridge University Press; 2011.

[13] Martins-Vale M, Pereira HP, Marina S, Ricou M. Conscientious objection and other motivations for refusal to treat in hastened death: a systematic review. Healthcare. 2023;11:2127. 10.3390/healthcare11152127. 37570368 10.3390/healthcare11152127 PMC10418655

[14] Triviño-Caballero R, Parra Jounou I, Roldán Gómez I, López de la Vieja MT. Causes for conscientious objection in medical aid in dying: a scoping review. CJB/RCB. 2023;6:102–14. 10.7202/1108007ar.

[15] Roehr B. Assisted dying around the world. BMJ. 2021;374:n2200. 10.1136/bmj.n2200. 34507973 10.1136/bmj.n2200

[16] Ministerio de Sanidad. Informe de evaluación anual 2022 sobre la prestación de ayuda para morir [2022 annual evaluation report on the medical assistance in dying benefit]. 2023. https://www.sanidad.gob.es/eutanasia/docs/InformeAnualEutanasia_2022.pdf. Accessed 6 May 2024.

[17] Derecho a Morir Dignamente. Primer informe sobre la evolución de la eutanasia en España de la Asociación Derecho a Morir Dignamente [First report on the evolution of euthanasia in Spain by the Right to Die with Dignity Association]. 2023. https://derechoamorir.org/wp-content/uploads/2023/06/Anexo-Informe-LORE-jun21-jun22.pdf. Accessed 6 May 2024.

[18] Vallès-Poch M, Parra Jounou I, Ortega-Lozano R, et al. End-of-life narratives of patients who request medical assistance in dying: a qualitative study protocol. Int J Qual Meth. 2023;22:16094069231202196. 10.1177/16094069231202196.

[19] Tong A, Sainsbury P, Craig J. Consolidated criteria for reporting qualitative research (COREQ): a 32-item checklist for interviews and focus groups. IJQHC. 2007;19:349–57. 10.1093/intqhc/mzm042. 10.1093/intqhc/mzm042 17872937

[20] Dempsey L, Dowling M, Larkin P, Murphy K. Sensitive interviewing in qualitative research. RINAH. 2016;39:480. 10.1002/nur.21743. 10.1002/nur.21743 27434172

[21] Ministerio de Sanidad. Informe anual 2021 de la prestación de ayuda para morir. [2021 annual report on the medical assistance in dying benefit]. 2022. https://www.sanidad.gob.es/eutanasia/docs/InformeAnualEutanasia_2021.pdf. Accessed 6 May 2024.

[22] Trejo-Gabriel-Galán JM. Variabilidad geográfica del número de eutanasias en España un año después de su legalización [Geographical variability of the number of euthanasias in Spain one year after its legalization]. Gac Sanit. 2023;37:102251. 10.1016/j.gaceta.2022.102251. 36099697 10.1016/j.gaceta.2022.102251

[23] Naeem M, Ozuem W, Howell K, Ranfagni S. A step-by-step process of thematic analysis to develop a conceptual model in qualitative research. Int J Qual Methods. 2023;22:16094069231205788. 10.1177/16094069231205789.

[24] Braun V, Clarke V. What can ‘thematic analysis’ offer health and wellbeing researchers? Int J Qual Stud Health Well-being. 2014;9:26152. 10.3402/qhw.v9.26152. 25326092 10.3402/qhw.v9.26152 PMC4201665

[25] Health Canada. Fourth Annual Report on Medical Assistance in Dying in Canada 2022. 2023. https://www.canada.ca/en/health-canada/services/publications/health-system-services/annual-report-medical-assistance-dying-2022.html. Accessed 6 May 2024.

[26] Generalitat de Catalunya. Informe anual sobre la aplicación de la Ley orgánica de regulación de la eutanasia durante el año 2022 en Cataluña [ Annual report on the application of the Organic Law regulating euthanasia during the year 2022 in Catalonia]. 2023. https://canalsalut.gencat.cat/web/.content/_Professionals/Consells_comissions/comissio-garantia-i-avaluacio-catalunya/informes/memoria-pram-es-22.pdf. Accessed 6 May 2024.

[27] Organización Médica Colegial de España-Consejo General de Colegios Oficiales de Médicos. Código de Deontología Médica [Deontological Medical Code]. 2022. https://www.cgcom.es/sites/main/files/minisite/static/828cd1f8-2109-4fe3-acba-1a778abd89b7/codigo_deontologia/. Accessed 6 May 2024.

[28] Word Medical Association. WMA International Code of Medical Ethics. 2022. https://www.wma.net/policies-post/wma-international-code-of-medical-ethics/. Accessed 6 May 2024.

[29] Piili RP, Louhiala P, Vänskä J, et al. Ambivalence toward euthanasia and physician-assisted suicide has decreased among physicians in Finland. BMC Med Ethics. 2022;23:71. 10.1186/s12910-022-00810-y. 35820881 10.1186/s12910-022-00810-y PMC9275272

[30] Cayetano-Penman J, Malik G, Whittall D. Nurses’ perceptions and attitudes about euthanasia: a scoping review. J Holist Nurs. 2021;39:66–84. 10.1177/0898010120923419. Erratum.In:JHolistNurs.2022;40:NP1-NP5. 32448052 10.1177/0898010120923419

[31] Patel T, Christy K, Grierson L, et al. Clinician responses to legal requests for hastened death: a systematic review and meta-synthesis of qualitative research. BMJ SP Care. 2021;11:59–67. 10.1136/bmjspcare-2019-002018. 10.1136/bmjspcare-2019-002018 32601150

[32] Gamondi C, Borasio GD, Oliver P, et al. Responses to assisted suicide requests: an interview study with Swiss palliative care physicians. BMJ SP Care. 2019;9:e7. 10.1136/bmjspcare-2016-001291. 10.1136/bmjspcare-2016-001291 28801317

[33] Voorhees JR, Rietjens JAC, van der Heide A, Drickamer MA. Discussing physician-assisted dying: physicians’ experiences in the United States and the Netherlands. Gerontologist. 2014;54:808–17. 10.1093/geront/gnt087. 24000266 10.1093/geront/gnt087

[34] Velasco Sanz TR, Cabrejas Casero AM, Rodríguez González Y, et al. Opinions of nurses regarding euthanasia and medically assisted suicide. Nurs Ethics. 2022;29:1721–38. 10.1177/09697330221109940. 35786045 10.1177/09697330221109940

[35] Barnes M, Brannelly T, Ward L, Ward N, editors. Ethics of care. Critical advances in international perspective. Bristol: Policy Press; 2015.

[36] Gilligan C. In a different voice: Psychological Theory and Women’s Development. Harvard, Mass: Harvard University Press; 1982.

[37] Koggel CM. Perspectives on Equality: Constructing a Relational Theory. New York: Rowman and Littlefield; 1998.

[38] Mackenzie C. Relational autonomy. In: Hall KQ, Ásta, editors. The Oxford handbook of feminist philosophy. Oxford: Oxford University Press; 2021. p. 374–84.

[39] Walker MU. Moral understandings. A feminist study in ethics. 2nd ed. New York: Routledge; 2007.

[40] Gómez-Vírseda C, de Maeseneer Y, Gastmans C. Relational autonomy: what does it mean and how is it used in end-of-life care? A systematic review of argument-based ethics literature. BMC Med Ethics. 2019;20:76. 10.1186/s12910-019-0417-3. 31655573 10.1186/s12910-019-0417-3 PMC6815421

[41] Dierickx S, Cohen J. Medical assistance in dying: research directions. BMJ Support Palliat Care. 2019;9:370–2. 10.1136/bmjspcare-2018-001727. 10.1136/bmjspcare-2018-001727 31676664

[42] Shaw J, Wiebe E, Nuhn A, et al. Providing medical assistance in dying: practice perspectives. Can Fam Phys. 2018;64:e394–9. PMC6135115 30209113

[43] Dinwiddie SH. Physician-assisted suicide: epistemological problems. Med Law. 1992;11:345–52. 1484459

[44] Bouthillier ME, Opatrny L. A qualitative study of physicians’ conscientious objections to medical aid in dying. Palliat Med. 2019;33:1212–20. 10.1177/0269216319861921. 31280666 10.1177/0269216319861921

[45] Buchbinder M, Brassfield ER, Mishra M. Health care providers’ experiences with implementing medical aid-in-dying in Vermont: a qualitative study. J Gen Intern Med. 2019;34:636–41. 10.1007/s11606-018-4811-1. 30684201 10.1007/s11606-018-4811-1 PMC6445925

[46] Ten Cate K, van Tol DG, van de Vathorst S. Considerations on requests for euthanasia or assisted suicide; a qualitative study with Dutch general practitioners. Fam Pract. 2017;34:723–9. 10.1093/fampra/cmx041. 28486577 10.1093/fampra/cmx041

[47] Verdaguer M, Beroiz-Groh P, Busquet-Duran X, et al. La ley de eutanasia y experiencias profesionales: tensiones en la práctica clínica [The euthanasia law and professional experiences: tensions in clinical practice]. Gac Sanit. 2024;10.1016/j.gaceta.2024.102373 10.1016/j.gaceta.2024.102373 38472012

[48] Andrade C. The inconvenient truth about convenience and purposive samples. Indian J Psychol Med. 2021;43:86. 10.1177/0253717620977000. 34349313 10.1177/0253717620977000 PMC8295573

