# Correction: For, against, and beyond: healthcare professionals’ positions on medical assistance in dying in Spain

**DOI:** 10.1186/s12910-024-01097-x

**Published:** 2024-09-23

**Authors:** Iris Parra Jounou, Rosana Triviño‑Caballero, Maite Cruz‑Piqueras

**Affiliations:** 1 Department of Philosophy, Universitat Autònoma de Barcelona, Bellaterra, Spain; 2 Department of Public Health and Maternal‑Child Health‑Faculty of Medicine, Universidad Complutense de Madrid, Madrid, Spain; 3 Escuela Andaluza de Salud Pública, Granada, Spain

**BMC Medical Ethics (2024) 25:69**

10.1186/s12910-024-01069-1

The funding information section in the article [1] contained an error in formatting. The corrected funder acknowledgment should be as follows:

Funding

This work has been developed in the framework of the following research projects: INEDYTO (PID2020-118729RB-I00), funded by MICIU/AEI/10.13039/501100011033; CONFINES (FD9/21_03), funded by the Programa de Ayudas a Proyectos de Investigación Científica 2021 (BBVA Foundation), and SOLBIO (PID2019-105422 GB-I00), funded by the Spanish Ministry of Science and Innovation. Iris Parra Jounou thanks the funding of the Spanish Research Agency (PID2019-105422 GB-I00).

The original article has been corrected.
