# Supplementary material for: For, against, and beyond: healthcare professionals’ positions on Medical Assistance in Dying in Spain
Source: BMC Med Ethics. 2024 Jun 14;25:69. doi: 10.1186/s12910-024-01069-1 (PMC11177400; doi:10.1186/s12910-024-01069-1)
Supplement: Supplementary file 1 — Supplementary Material 1. [file 12910_2024_1069_MOESM1_ESM.docx]

**Interview script**

1. **Introduction**

- Presentation of the research project: experience and expectations about the practice of Medical Assistance in Dying; beliefs and knowledge about Conscientious Objection and its compatibility with Medical Assistance in Dying.

- Approximate duration of the interview (1 hour).

- Information and consent process, with particular emphasis on confidentiality; access to information only by the research team; coding of information once collected; the possibility of withdrawal at any time; and the freedom to comment on any relevant aspect.

- Permission to record: record CI or collect it in writing.

1. **Approach to interviewee profile**

- What does your work consist of? What are your years of experience? What is the profile of patients you care for? Do you have any training in bioethics? Do you know your center's Health Care Ethics Committee *(experience with it)*?

1. **Ethical conflicts at the end of life**

- Ethical conflicts that you might have or have had when dealing with end-of-life scenarios *(requests for medical assistance in dying, relief of suffering, refusal of treatment, treatment withdrawal, adequacy of life support measures...)*.

1. **Evaluation of the Spanish Law Regulating Euthanasia (LORE)**

The end-of-life scenario often raises ethical conflicts for professionals, patients, and relatives. In this sense, approving the Spanish Law regulating Euthanasia (LORE) introduces new elements to the debate and raises certain controversies. Regarding this issue:

- In general terms, how do you assess the introduction of a law regulating Medical Assistance in Dying?

- According to you, what do you think have been the main problems in its implementation/development *(look into possible conscientious objection)*?

- In your experience, are health professionals prepared to provide this benefit, which is guaranteed by law?

- In your work environment, what "atmosphere" has the approval of the law produced? What comments has this issue raised among other colleagues?

- Have you received any information from your superiors/management or institution regarding medical assistance in dying?

- Have you received any request for medical assistance in dying? *(If so, please describe the case: type of patient, if and when you knew him/her/them, relationship with other professionals, difficulties of the process, evaluation of the experience,...)*.

- What would happen if you received a request for medical assistance in dying? How would you act? How do you imagine the process would be? And the relationship with other professionals, the patient, and the family? Would you have any doubts?

1. **Conscientious objection**

- How would you define Conscientious Objection? Do you think it is an ethically acceptable reason for not providing Medical Assistance in Dying?

- Regarding the possibility of objecting, do you know who can object? Do you know the procedure to object? What is your opinion on that?

- Would you consider objecting in any case? If so, in what kind of situations? Why?

- Do you know of any colleague who has declared himself/herself/themselves a conscientious objector to Medical Assistance in Dying? Do you know of any colleague who has objected to a specific case? Could you describe the situation? Do you know if he/she/they had registered his/her/their objection?

- What do you think is the rationale/motivation leading to conscientious objection?

Do you know of people who use Conscientious Objection for reasons other than their intimate, personal convictions? If so, which reasons would they be? Why would they do it? *(Ask about motivations other than conscientious objections: possible pressures from the team or from the healthcare facility, fear of stigma, overload of work, emotional burden, etc.).*

The process of Medical Assistance in Dying has different phases (information, first written request, being a “responsible physician," second written request, being a “consulting physician," referring the patient to another professional, etc.). In your opinion, where in that process would it be ethically acceptable to object?

- Do you consider that Conscientious Objection could be an obstacle to providing Medical Assistance in Dying? If so, in what way?

1. **Proposals for improvement**

To conclude, I would like to discuss some final questions:

- According to you, what could the Healthcare System do to better reconcile Conscientious Objection with the right to Medical Assistance in Dying recognized by law? And on the part of the professionals?

- In which ways could the study we are conducting be of help?

Thank you for your attention. If there is anything we have yet to cover that I have not asked you about but that you would like to share...
